# Association of arterial stiffness with incident atrial fibrillation: a cohort study

**DOI:** 10.1186/s12872-021-02057-8

**Published:** 2021-05-20

**Authors:** Zakaria Almuwaqqat, J.Neka S. Claxton, Faye L. Norby, Pamela L. Lutsey, Jingkai Wei, Elsayed Z. Soliman, Lin Y. Chen, Kunihiro Matsushita, Gerardo Heiss, Alvaro Alonso

**Affiliations:** 1 Department of Epidemiology, Rollins School of Public Health, Emory University, Atlanta, GA USA; 2 Department of Medicine, Division of Cardiology, Emory School of Medicine, 1364 Clifton Rd NE, Atlanta, GA 30322 USA; 3 Division of Epidemiology & Community Health, School of Public Health, University of Minnesota, Minneapolis, MN USA; 4 Department of Epidemiology and Biostatistics, George Washington University, Washington, D.C USA; 5 Epidemiological Cardiology Research Center, Department of Epidemiology and Prevention, Wake Forest School of Medicine, Winston-Salem, NC USA; 6 Cardiovascular Division, Department of Medicine, University of Minnesota Medical School, Minneapolis, MN USA; 7 Department of Epidemiology and the Welch Center for Prevention, Epidemiology and Clinical Research, Johns Hopkins Bloomberg School of Public Health and the Division of General Internal Medicine, Department of Medicine, Johns Hopkins University, Baltimore, MD USA; 8 Department of Epidemiology, Gillings School Public Health, University of North Carolina, Chapel Hill, NC USA

**Keywords:** Arterial stiffness, Atrial fibrillation, Hypertension

## Abstract

**Background:**

Stiff arteries increase left ventricular (LV) end-systolic workload, leading over time to left atrial and ventricular remodeling, and providing the substrate for atrial fibrillation (AF) development. We investigated if carotid femoral pulse wave velocity (cfPWV), a measure of central arterial stiffness, is associated with incident AF.

**Methods:**

In 20112013, cfPWV was measured in 3882 participants of the Atherosclerosis Risk in Communities Cohort Study (ARIC) without prevalent AF. Participants were followed through 2017 for the incidence of AF. Individuals were categorized in cfPWV quartiles based on visit measurements. Multivariable Cox regression models were used to evaluate the association of cfPWV with incident AF.

**Results:**

Mean age was 75years (SD 5), 60% were female and 20% were African American. Over a median follow-up of 5.5years we identified 331 incident cases of AF. cfPWV demonstrated U-shaped associations with AF risk. In models adjusted for age, race, center, sex, education levels, and hemodynamic and clinical factors, hazard ratios (HR) of AF for participants in the first, third and fourth quartiles were 1.49 (95% CI 1.06, 2.10), 1.59 (1.14, 2.10), and 1.56(1.10, 2.19), respectively, compared to those in the second quartile.

**Conclusion:**

Among community-dwelling older adults, low and high central arterial stiffness is associated with AF risk.

**Supplementary Information:**

The online version contains supplementary material available at 10.1186/s12872-021-02057-8.

## Background

Atrial fibrillation (AF) is the most common sustained cardiac arrhythmia and confers increased risks of morbidity and mortality [1]. Hypertension is considered among the most commonly encountered risk factors for AF [2]. Arterial stiffening is part of the substrate for sustained high blood pressure, which can lead in turn to increased left ventricular end-systolic workload, followed by ventricular remodeling [3]. Simultaneously, impaired ventricular diastolic function and increased atrial pressure might result in fibrosis and electrical remodeling in the atrium and predispose to future AF [4]. Similarly, aging is thought to drive a disproportionate rise in central arterial stiffness, even among subjects at low cardiovascular risk, with a concomitant significant increase in AF risk [5, 6]. There is evidence that central arterial stiffness measures are associated with biomarkers of myocardial stress among older adults without cardiac disease and incident heart failure in a U-shaped pattern [7]. However, the doseresponse pattern between these measures and AF risk is not clear. Pulse wave velocity (PWV) is considered the gold standard for non-invasive assessment of arterial stiffness across different ages [8]. Prior studies evaluating the association of arterial stiffness, measured by PWV, with AF risk were done in racially homogeneous populations, did not focus on elderly individuals specifically and lacked control over markers of left atrial overload [9, 10]. Therefore, in this analysis, we examined associations of segment-specific PWV measures with the incidence of AF in a large cohort of community-dwelling black and white older adults without a history of prevalent AF in the Atherosclerosis Risk in Communities (ARIC) study.

## Methods

### Study population

**Fig. 1:**
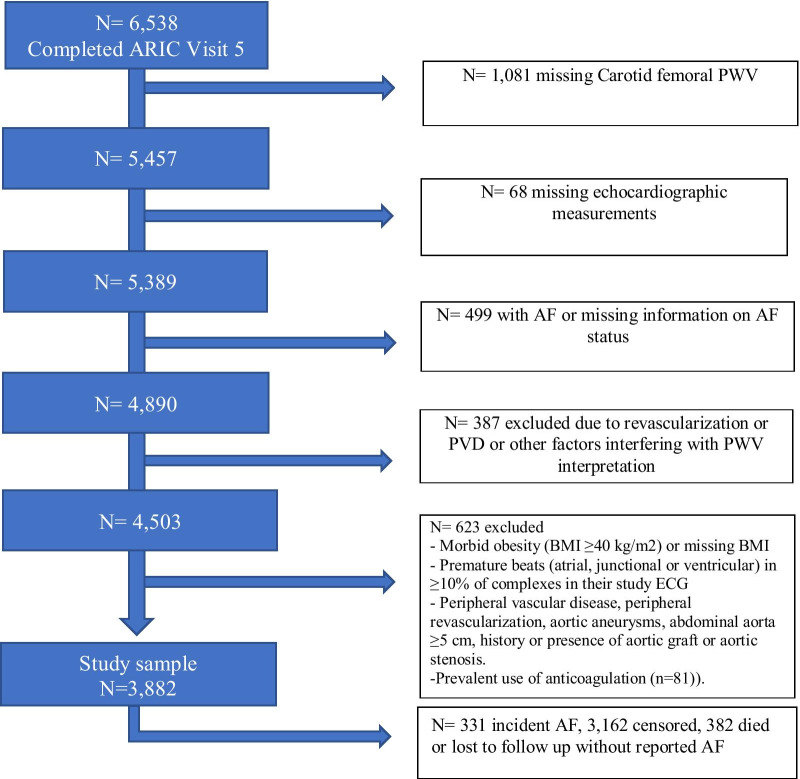
Sample inclusion flowchart

The ARIC study is a prospective epidemiologic cohort conducted in four U.S. communities: Washington County, Maryland; Jackson, Mississippi; selected Minneapolis suburbs, Minnesota; and Forsyth County, North Carolina [11]. A detailed description of the design and objectives of the ARIC cohort study has been published [11]. Approximately 4000 individuals aged 4564 were recruited from each ARIC center. In 19871989, a baseline examination (visit 1) was completed in 15,792 individuals (55% women, 27% blacks). Participants were then followedup regularly every three years until 1998, with the second exam (visit 2) occurring in 19901992, the third (visit 3) in 19931995, and the fourth (visit 4) in 19961998. A fifth exam (visit 5) occurred in 20112013, a sixth exam (visit 6) in 20162017 and a seventh exam in 20182019. For this analysis, we included participants with PWV measurements at visit 5. Of these, we excluded participants who had developed AF by visit 5 regardless of heart failure history (Fig.1). Of these, we excluded participants who had developed AF by visit 5 (Fig.1). We also excluded those with a race other than white or African American and the few African Americans in the Minnesota and Washington County cohorts, morbid obesity (BMI40kg/m^2^) or missing BMI; those with premature beats (atrial, junctional or ventricular) in10% of complexes in their study ECG; and those with peripheral vascular disease, peripheral revascularization, aortic aneurysms, abdominal aorta with dilation5cm, presence aortic graft or aortic stenosis [12]. Finally, we also excluded subjects with the prevalent use of anticoagulation (n=81), as they may have prevalent AF not identified by the study to eliminate potential misclassification. The ARIC study is performed in perthe Declaration of Helsinki and has been approved by Institutional Review Boards (IRB) and ethics committees at all participating institutions: theUniversity of North Carolina at Chapel Hill IRB, Johns Hopkins University IRB, University of Mississippi Medical Center IRB, and University of Minnesota IRB [11]. Study participants provided written informed consent at all study visits. All methods were carried out in accordance with relevant guidelines and regulations.

### Assessment of PWV

The protocol of PWV measurements in ARIC has been described previously [13]. Carotid-femoral PWV (cfPWV) was obtained using an automatic vascular screening device (VP-1000 Plus; Omron Healthcare, Kyoto, Japan) [14]. Carotid and femoral arterial pressure waveforms were acquired in the supine position after 5min of rest by applanation tonometry sensors attached to the left common carotid artery (via neck collar) and left femoral artery (via elastic tape around the hip) [13]. The minimum data acquisition was 30s. The set of measurements was repeated after a brief rest period (5min). The distance between two arterial sites (carotid and femoral for example) divided by the time the wave takes to travel that distance was used to calculate the PWV, with higher values indicating greater arterial stiffness [15]. Distance for cfPWV was measured with a segmometer (Rosscraft, Surray, Canada) and calculated as the distance between the carotid to the femoral distance minus the suprasternal notch to the carotid. In addition to cfPWV, brachial-ankle PWV (baPWV) and femoral-ankle PWV (PWV) were automatically calculated by the VP-1000 Plus device using height-based formulas [16].

Study personnel were centrally trained, and a quality control program was established by which a random sample of 40 records per month, stratified by center, were reviewed. Based on this, technicians received feedback on data quality and completeness. The repeatability of cfPWV measurements taken 48weeks apart (assessed with the intraclass correlation coefficient) was 0.70 (95%CI 0.590.81), suggesting adequate validity [17]. For our primary analysis, cfPWV was considered to reflect central arterial stiffness, while faPWV was considered to represent peripheral arterial stiffness, and baPWV was considered as a mixed measure of central and peripheral stiffness. For faPWV and baPWV the higher value of the left and right PWV was used for our primary analysis.

Blood samples were drawn at visit 5 and laboratory tests were performed according to a common protocol by trained technicians at each of the ARIC field centers [12, 13]. Circulating N-terminal B-type natriuretic peptide (NT-proBNP) at visit 5 was measured on the Roche Elecsys 2010 Analyzer (Roche Diagnostics, Indianapolis, IN 46250) using immunoassay methods [18, 19].

### Determination of incident AF

We used three methods to identify AF cases in the ARIC cohort that have been described previously [20]. In summary, first, trained abstractors collected information from participants hospitalization identified by follow-up phone calls and surveillance of local hospitals, including all discharge codes that are relevant for AF or atrial flutter. AF was deemed as present if ICD-9-CM codes 427.31 (AF) or 427.32 (atrial flutter) or ICD-10-CM codes I48.x were listed in any given hospitalization. We excluded AF cases occurring in the context of cardiac surgery. Second, 12-lead electrocardiograms (ECGs) were obtained during study exams (visits 1 through 5), and data were transmitted electronically to the ARIC ECG reading center at EPICARE (Wake Forest School of Medicine, Winston-Salem, NC), using the GE Marquette 12-SL program (GE Marquette, Milwaukee, WI) for processing [20]. A computer algorithm identified the presence of AF or atrial flutter in the ECG. Those ECGs were further reviewed by a cardiologist to confirm the computer diagnosis of AF and also overread any rhythm disorder other than AF in electrocardiograms to reduce the possibility of missed or misreading episodes of AF. Given that visit 5 was the last visit in which ECGs were performed, this method was only used to determine prevalent AF. Lastly, AF diagnosis was identified from death certificates if ICD-9 code 427.3 or ICD-10 code I48 were listed as any cause of death. Overall, AF hospitalization represented 96% of all AF cases ascertained after visit 5. These methods of AF ascertainment have been validated previously in ARIC [20]. These methods have been validated in prior analyses with satisfactory validity and sensitivity [20].

### Ascertainment of other covariates

We considered the following covariates assessed in visit 5: sex, age, race, study center, education level (at visit 1), systolic blood pressure (SBP), diastolic blood pressure (DBP), smoking status, alcohol drinking status, diabetes history, heart failure (HF) history, myocardial infarction (MI) history, use of aspirin medications, use of statin medications, echocardiographic left ventricular ejection fraction (LVEF), and left atrial (LA) volume. Brachial artery pressure was measured in the sitting position after resting for 5min, using an Omron HEM907XL (Healthcare, Kyoto, Japan) oscillometric sphygmomanometer, with anaverage of 3 measurements used for analysis. Diabetes was defined as the use of anti-diabetic medication, or a self-reported physician diagnosis of diabetes, fasting blood glucose126mg/dl, or a non-fasting blood glucose200mg/dL. Information regarding sex, race, age, smoking status, and alcohol intake was self-reported. Medications taken in the prior 2weeks were brought to the clinic visit; the names of the medications were recorded. HF history was derived based on the Gothenburg criteria at visit 1 and from HF-related hospitalizations (ICD-9 codes for HF) during follow-up [21]. MI was defined based on a self-reported physician diagnosis of MI at baseline or evidence of old MI on ECG and events adjudicated during the follow-up [22].

### Statistical analysis

We modeled PWV measurements using restricted cubic splines to characterize the doseresponse associations with AF risk. This analysis used the median PWV value as the reference point for risk assessment. Based on the evidence of non-linearity in the association of cfPWV with AF incidence, we evaluated the risk of AF across categories of cfPWV defined using quartiles. In additional analyses, we assessed AF risk across quartiles of baPWV and faPWV. We calculated the cumulative incidence of AF accounting for the competing risk of death using the cumulative incidence function [23]. Cox proportional hazards models were used to calculate hazard ratios of developing AF and their 95% confidence intervals according to cfPWV quartiles, using the second quartile as the reference category. The time of follow-up was defined as days from visit 5 to AF incidence, death, lost to follow up, or December 31, 2017, whichever occurred earlier. We used the following two models with incremental adjustments to analyze the association of cfPWV with AF risk (1) Model 1: adjustment for age (continuous), sex (dichotomous), education level (grade school, high school but not graduate, high school graduate, vocational school, college, graduate school), race and ARIC study center; (2) Model 2: further adjusted for smoking (current, former, never), drinking (current, former, never), diabetes mellitus (dichotomous), history of MI (dichotomous), aspirin use (dichotomous), statin use (dichotomous), LVEF (continuous), DBP and SBP (continuous) and left atrial volume (continuous). We further adjusted for heart failure as a time-varying covariate and NT-proBNP levels at visit 5. Finally, we conducted analyses stratified by sex and race. Data analysis was conducted with SAS software version 9.4 [SAS Institute Inc., Cary, NC].

### Patient and public involvement

There was no direct patient involvement in study design, analysis, interpretation or writing.

## Results

The mean age for the study cohort of 3882 participants without AF at visit 5 was 75years, with women accounting for 60% and African Americans accounting for 20% of the analyzed cohort (Table 1). Increasing cfPWV was associated with advanced age, male sex, African American race, higher SBP, higher heart rate, higher prevalence of DM. LVEF and LA volume were similar across the four quartiles.

**Table 1:** Baseline characteristics of the ARIC cohort participants included in the study by quartiles of carotid-femoral pulse wave velocity (cfPWV), 20112013 (n=3882)

Variable	1st quartile	2nd quartile	3rd quartile	4th quartile
cfPWV, m/s	<9.5	9.511.2	11.2 -13.2	>13.2
N	972	971	974	965
Age, year	74 (4.6)	74 (4.6)	75 (4.9)	77(5.1)
Women, %	65.2%	60.5%	58.5%	56.9%
African American race, %	16.1%	16.7%	18.8%	28.5%
Completed high school, %	90.5%	89.2%	88.2%	83.3%
BMI, kg/m^2^	27.5 (4.3)	28.0 (4.4)	27.9 (4.3)	27.5 (4.7)
SBP, mmHg	122.4 (15.5)	127.2 (15.4)	132.5(16.6)	138.1 (18.4)
Heart Rate, bpm	62.6 (9.9)	63.8 (9.7)	65.8 (10.6)	67.4 (11.2)
Current smoker, %	7.4%	6.4%	5.9%	5.1%
Current alcohol Use, %	56.3%	55.6%	51.2%	41.7%
Aspirin Use, %	68.5%	69.5%	69.2%	65.3%
Statin Use, %	47.2%	49.3%	48.7%	53.9%
Diabetes, %	21.4%	25.3%	30.9%	39.4%
LVEF, %	65.9 (5.9)	66.0 (5.9)	65.7 (6.0)	65.3 (5.9)
LA Volume, mm^3^	46.2 (17.2)	45.7 (14.7)	46.5 (15.3)	46.1 (15.4)
History of HF, %	2.3%	1.9%	2.1%	2.9%
History of MI, %	7.4%	6.1%	8.0%	9.4%

ARIC, atherosclerosis risk in communities study; SD standard deviation; BMI, body mass index; SBP, systolic blood pressure; MI, myocardial infarction; HF, heart failure; LA, left atrial; MI, myocardial infarction; DM, diabetes mellitus; LVEF, left ventricular ejection fraction

Continuous variable given as mean (SD) and categorical variables given as %

**Fig. 2:**
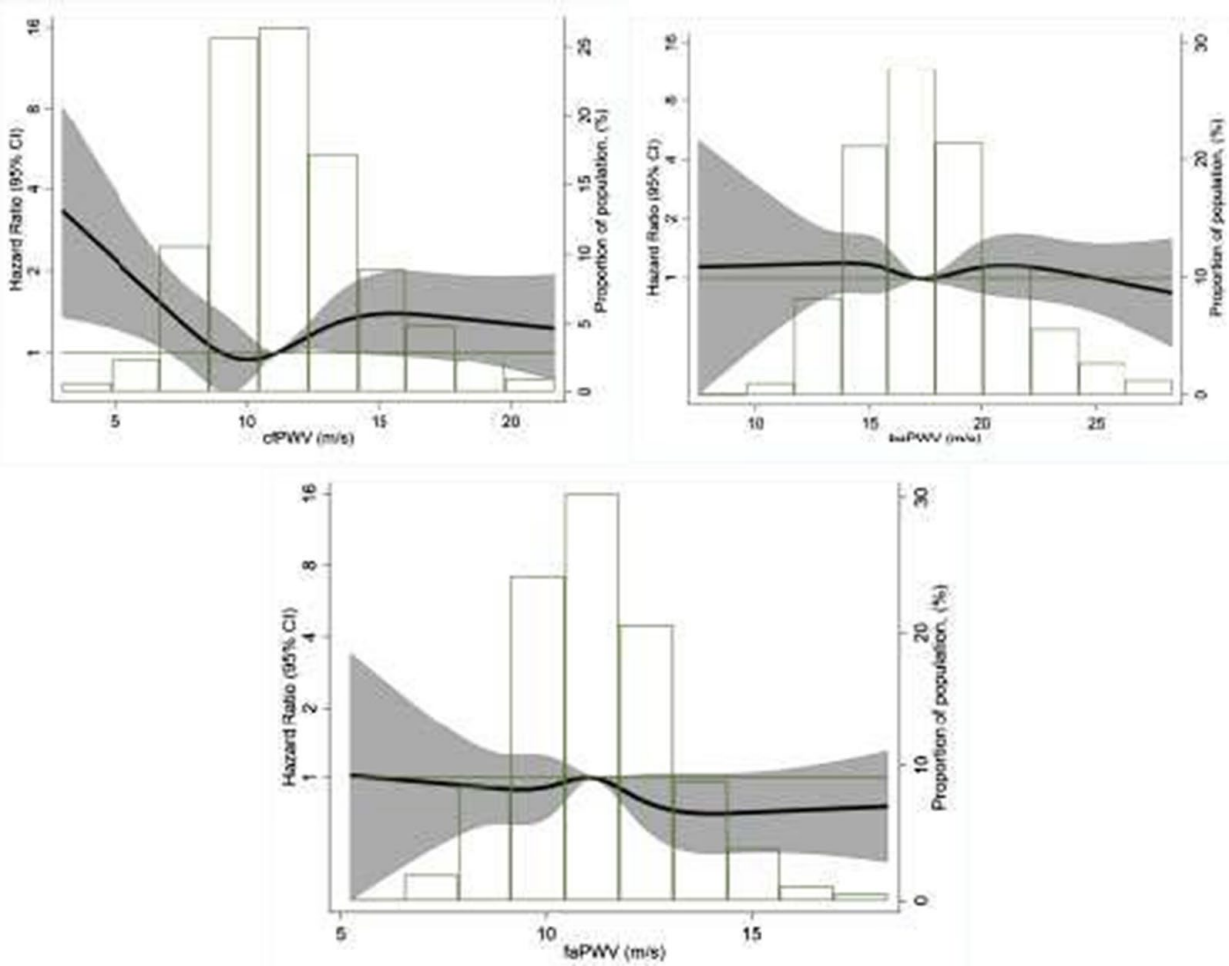
Age, sex, and race-adjusted hazard ratios and 95% confidence intervals of AF by cfPWV (top panel), baPWV (middle panel) and faPWV values (bottom panel). PWV measurements modeled as restricted cubic splines. The median value of PWV measurements was considered the reference (HR=1). The histograms represent the frequency distribution of PWV in the study sample. ARIC 20112017

**Fig. 3:**
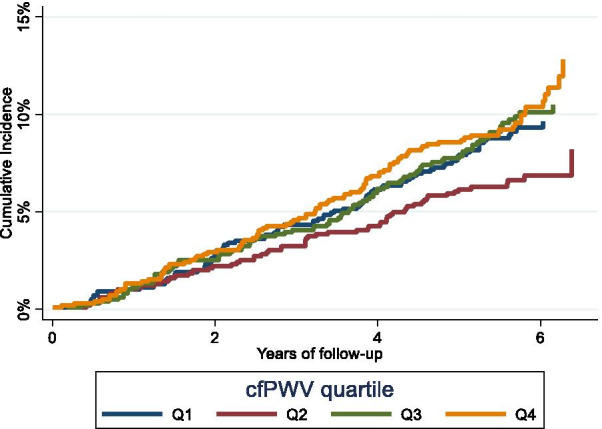
Cumulative incidence of AF, unadjusted, by quartiles of carotid-femoral pulse wave velocity after ARIC visit 5, considering death as a competing risk, ARIC 20112017

Over a median follow-up of 5.5years, we identified 331 incident cases of AF. 3162 censored at the end of follow-up while 382 died or lost to follow up without reported AF (Fig.1). Modeling cfPWV with a restricted cubic spline showed a U-shaped association with the incidence of AF (Fig.2). Based on the presence of this non-linear association, cfPWV was categorized and modeled in quartiles. Figure3 depicts the cumulative incidence of AF by quartiles of cfPWV considering death as a competing event, showing the lowest risk among participants in the second quartile. In minimally adjusted modes, participants with cfPWV in the first, third and fourth quartiles were at a greater risk of incident AF once adjusted for age, race, center, sex and education levels: hazard ratios (HR) for first, third and fourth quartiles were 1.60 (95% CI 1.14, 2.24), 1.56 (1.12, 2.17), 1.47 (1.05, 2.06), as compared to the second quartile (Table 2). After further adjustment for clinical and hemodynamic factors, results were similar [HR 1.49 (95% CI 1.06, 2.10), 1.59 (1.14, 2.10), 1.56 (1.10, 2.19), Table 2]. Additional adjustment for heart failure as a time-varying covariate or NT-proBNP did not modify the associations (Table 2, Models 3 and 4). Moreover, when we used the Fine and Gray model while considering death as a competing event, results were consistent (Table 2, Model 5).

**Table 2:** Association of carotid femoral pulse wave velocity indices with incident AF in the ARIC study, 20112017. (n=3882)

	PWV quartiles			
	1st quartile	2nd quartile	3rd quartile	4th quartile
cf PWV (m/s)	<9.5	9.511.1	11.213.2	>13.2
# Incident AF cases	181	57	93	96
Person-time (years)	5169	5153	5073	5041
HR (95% CI)				
Model 1	1.60 (1.14, 2.24)	Ref	1.56 (1.12, 2.17)	1.47 (1.05, 2.06)
Model 2	1.49 (1.06, 2.10)	Ref	1.59 (1.14, 2.10)	1.56 (1.10, 2.19)
Model 3	1.51 (1.07, 2.13)	Ref	1.63 (1.16, 2.28)	1.68 (1.18, 2.38)
Model 4	1.48 (1.05, 2.10)	Ref	1.64 (1.17, 2.30)	1.53 (1.8, 2.18)
Model 5*	1.51 (1.07, 2.13)	Ref	1.58 (1.12, 2.21)	1.58 (1.11, 2.25)

Abbreviation: baPWV, brachial-ankle pulse wave velocity; cfPWV, carotid-femoral pulse wave velocity, CI; confidence interval; faPWV: femoral-ankle pulse wave velocity; HR, hazard ratio

Model 1: Cox regression model adjusted for sex, age, center, race, education level

Model 2: Cox regression model adjusted for sex, age, center, race, education level, systolic blood pressure, diastolic blood pressure, alcohol use, cigarettes smoking, history of myocardial infarction, history of heart failure, body mass index, aspirin, statin, hypertension, diabetes mellitus, left ventricular ejection fraction and left atrial volume

Model 3: Model 2 further adjusted for heart failure as a time dependent covariate

Model 4: Model 2 further adjusted for NT-pro B-type natriuretic peptide

^*^Model 5: Fine and Gray competing risks model adjusted for variables in model 2, with death considered as a competing risk

In sex and race-stratified analyses, we found that associations of cfPWV with AF risk were not significantly different between men and women (P for interaction=0.40) and among whites and African Americans (P for interaction=0.60) (Additional file 1: Table S1).

We did not observe associations of markers of peripheral arterial stiffness (baPWV and faPWV) with the risk of AF (Fig.2). Compared to participants in the second quartile of baPWV, the HRs (95%CI) of AF were 1.05 (0.76, 1.44, 0.89 (0.65, 1.22, and 1.07 (0.79, 1.45) in the 1st, 3rd and 4th quartiles, respectively (Table 3, Model 1). Further adjustment for clinical and hemodynamic factors did not meaningfully impact the results (Table 3, Model 2). Similarly, faPWV was not associated with AF risk (Table 3).

**Table 3:** Association of peripheral pulse wave velocity indices with incident AF in the ARIC study, 20112017. (n=3882)

ba PWV (m/s)	<15.4	15.417.3	17.319.6	>47.1
# Incident AF cases	75	79	76	101
Person-time (years)	5062	5187	5155	5033
HR (95% CI)				
Model 1	1.05 (0.76, 1.44)	Ref	0.89 (0.65, 1.22)	1.07 (0.79, 1.45)
Model 2	0.99 (0.72, 1.37)	Ref	0.95 (0.69, 1.30)	1.25 (0.91, 1.72)

fa PWV (m/s)	<10.0	10.011.1	11.112.3	>37.2
# Incident AF cases	87	86	85	73
Person-time (years)	4974	5147	5086	5230
HR (95% CI)				
Model 1	1.05 (0.78, 1.42)	Ref	0.97 (0.71, 1.30)	0.77 (0.56, 1.05)
Model 2	1.00 (0.72, 1.36)	Ref	1.06 (0.78, 1.44)	0.90 (0.65, 1.25)

Model 1: Cox hazaredregression modeladjusted for sex, age, center, race, education level

Model 2: Cox hazardregression modeladjusted for sex, age, center, race, education level, systolic blood pressure, diastolic blood pressure, alcohol use, cigarettes smoking, history of myocardial infarction, history of heart failure, body mass index, aspirin, statin, hypertension, diabetes mellitus, left ventricular ejection fraction and left atrial volume

## Discussion

Our study demonstrates a U-shaped pattern between central arterial stiffness as measured by cfPWV and incidence of AF during a follow-up of 5.5years among ARIC community-dwelling older adults. The association remained even after adjustment for clinical risk factors, hypertension, LVEF, and LA enlargement. The association was similar in men and women, whites, and blacks. We did not find associations between markers of peripheral arterial stiffness (faPWV and baPWV) and AF risk.

We observed that individuals with higher cfPWV presented an increased risk of AF, an observation that has biological plausibility. Despite the evidence for a potential bidirectional relationship between arterial stiffening and hypertension, stiff arteries can be part of the substrate for high systemic blood pressure leading to adverse left ventricular remodeling [24]. Adverse left ventricular changes, including concentric hypertrophy and altered geometry, are known to be associated with structural and functional changes in the left atrium that ultimately increase AF risk [25]. In fact, animal models have shown that atrial fibroblasts have greater fibrotic potential than their ventricular counterparts, suggesting that the left atrium is more sensitive to adverse vascular remodeling than the left ventricle [26]. At the same time, measures of endothelial dysfunction, such as flow-mediated dilation, have been associated with AF [10]. Thus, it is possible that arterial stiffness is a marker of cumulative vascular injury and left atrial remodeling that will ultimately signal future AF development.

Prior studies have shown that pulse pressure, as another measure of arterial stiffness, correlates with agreater risk of AF [2]. Those results were validated by other studies showing that pulse wave velocity can predict AF in sex and age-adjusted models [10]. However, the association was non-significant after adjustment for covariates while considering cfPWV as a continuous variable. Moreover, the prior studies were limited by the lack of control over echocardiographic markers of left atrial overload, including LVEF and LA volume.

In this study, we show for the first time that the association of central PWV with AF follows a non-linear pattern, with a higher risk of AF for those with low or high central PWV. These results are consistent with findings from prior studies showing that cfPWV is associated with cardiac biomarkers of myocardial stress and incident heart failure in aU-shaped pattern [12]. A similar trend, albeit non-significant, has been previously reported between cfPWV and heart failure with preserved ejection fraction in the health ABC study [27]. However, exact mechanisms explaining the association between low cfPWV and AF risk remain unclear [28]. One possible mechanism is that low cfPWV may point to subclinical cardiovascular dysfunction decreasing cfPWV and contributing to the AF risk in this group. Studies have shown that cardiac stroke volume and overall cardiac output correlates with cfPWV and thus heart failure, a known risk factor for AF, manifesting with subclinical low cardiac output that may lead to spuriously low cfPWV [29]. However, the lack of attenuation in the association between cfPWV and AF after adjustment for NT-proBNP and heart failure as a time-varying covariate does not support this hypothesis. Future studies should replicate this U-shaped association and evaluate underlying mechanisms.

Our study has several strengths. Firstly, the ARIC study includes a community-based and diverse population with measures of arterial stiffness, biomarkers, and outcomes. Secondly, our study had an extensive quality control program to ensure the replicability of results. Moreover, ARIC utilized centrally trained personnel to perform measurements. Random samples of PWV were taken from all centers to confirm measurement repeatability. Our study, however, also has limitations. Firstly, our approach for AF ascertainment probably led to information bias due to missing asymptomatic cases as well as those managed exclusively in the outpatient setting. However, we have conducted a previous validation study in the ARIC cohort showing that this method of AF ascertainment has adequate sensitivity, specificity, and predictive values [20]. We also excluded patients with anticoagulation use which might be prescribed for possible subclinical AF. Moreover, other large epidemiologic studies have used similar approaches, also with good validity [30]. Secondly, our analysis was limited to individuals who completed an ARIC clinic visit 5 and had no major arrhythmias or revascularization. Moreover, our results are mainly derived from community-based older individuals. Thus, our results may not be generalizable to younger individuals and those who died prior to visit 5 or chose not to take part. Finally, being an observational study, there remains the potential for residual and unmeasured confounding. Still, our analysis adjusted for the main risk factors for AF, reducing the likelihood of confounding being a major explanation for our findings.

## Conclusion

In conclusion, arterial stiffness is independently associated with AF risk. Among older ARIC participants, central arterial stiffness displayed a U-shaped association with AF risk. Older adults with high or low arterial stiffness as measured by cfPWV might be at high risk of AF development. Mechanisms underlying this association deserve further study so potential strategies for AF prevention can be developed.

## Supplementary Information

**Additional file 1. Table S1**. Associations of Carotid-Femoral Pulse Wave Velocity (cfPWV) quartiles with incident AF in the ARIC cohort by sex and race, 20112017.

## Data Availability

The datasets used and analyzed during the current study available from the corresponding author on reasonable request.

## Abbreviations

ARIC: Pulse wave velocity
AF: Atrial fibrillation
baPWV: Pulse wave velocity
cfPWV: Pulse wave velocity
CI: Confidence interval
DBP: Diastolic blood pressure
DM: Diabetes Mellitus
ECG: Electrocardiography
faPWV: Femoral-ankle pulse wave velocity
HF: Heart failure
HR: Hazard ratio
ICD: International classification of disease
LA: Left atrium
LVEF: Left ventricular Ejection Fraction
MI: Myocardial infarction
NT-proBNP: N-terminal B-type natriuretic peptide
PWV: Pulse wave velocity
SBP: Systolic blood pressure

## References

[1] Benjamin EJ, Muntner P, Alonso A, Bittencourt MS, Callaway CW, Carson AP, Chamberlain AM, Chang AR, Cheng S, Das SR, Delling FN, Djousse L, Elkind MSV, Ferguson JF, Fornage M, Jordan LC, Khan SS, Kissela BM, Knutson KL, Kwan TW, Lackland DT, Lewis TT, Lichtman JH, Longenecker CT, Loop MS, Lutsey PL, Martin SS, Matsushita K, Moran AE, Mussolino ME, O'Flaherty M, Pandey A, Perak AM, Rosamond WD, Roth GA, Sampson UKA, Satou GM, Schroeder EB, Shah SH, Spartano NL, Stokes A, Tirschwell DL, Tsao CW, Turakhia MP, VanWagner LB, Wilkins JT, Wong SS, Virani SS, American Heart Association Council on Epidemiology and Prevention Statistics Committee and Stroke Statistics Subcommittee (2019). Heart Disease and Stroke Statistics-2019 update: a report from the American Heart Association. Circulation.

[2] Mitchell GF, Vasan RS, Keyes MJ, Parise H, Wang TJ, Larson MG, D'Agostino RB, Kannel WB, Levy D, Benjamin EJ (2007). Pulse pressure and risk of new-onset atrial fibrillation. JAMA.

[3] Hundley WG, Kitzman DW, Morgan TM, Hamilton CA, Darty SN, Stewart KP, Herrington DM, Link KM, Little WC (2001). Cardiac cycle-dependent changes in aortic area and distensibility are reduced in older patients with isolated diastolic heart failure and correlate with exercise intolerance. J Am Coll Cardiol.

[4] Dzeshka MS, Lip GY, Snezhitskiy V, Shantsila E (2015). Cardiac Fibrosis in Patients With Atrial Fibrillation: Mechanisms and Clinical Implications. J Am Coll Cardiol.

[5] Mitchell GF, Parise H, Benjamin EJ, Larson MG, Keyes MJ, Vita JA, Vasan RS, Levy D (2004). Changes in arterial stiffness and wave reflection with advancing age in healthy men and women: the Framingham Heart Study. Hypertension.

[6] Lakatta EG, Levy D (2003). Arterial and cardiac aging: major shareholders in cardiovascular disease enterprises: Part II: the aging heart in health: links to heart disease. Circulation.

[7] Kim ED, Ballew SH, Tanaka H, Heiss G, Coresh J, Matsushita K (2019). Short-term prognostic impact of arterial stiffness in older adults without prevalent cardiovascular disease. Hypertension.

[8] Laurent S, Cockcroft J, Van Bortel L, Boutouyrie P, Giannattasio C, Hayoz D, Pannier B, Vlachopoulos C, Wilkinson I, Struijker-Boudier H, on behalf of the European Network for Non-invasive Investigation of Large Arteries (2006). Expert consensus document on arterial stiffness: methodological issues and clinical applications. Eur Heart J..

[9] Chen LY, Leening MJ, Norby FL, Roetker NS, Hofman A, Franco OH, Pan W, Polak JF, Witteman JC, Kronmal RA, Folsom AR, Nazarian S, Stricker BH, Heckbert SR, Alonso A (2016). Carotid intima-media thickness and arterial stiffness and the risk of atrial fibrillation: the Atherosclerosis Risk in Communities (ARIC) Study, Multi-Ethnic Study of Atherosclerosis (MESA), and the Rotterdam Study. J Am Heart Assoc..

[10] Shaikh AY, Wang N, Yin X, Larson MG, Vasan RS, Hamburg NM, Magnani JW, Ellinor PT, Lubitz SA, Mitchell GF, Benjamin EJ, McManus DD (2016). Relations of arterial stiffness and brachial flow-mediated dilation with new-onset atrial fibrillation: the Framingham Heart Study. Hypertension.

[11] The ARIC Investigators (1989). The Atherosclerosis Risk in Communities (ARIC) study: design and objectives. Am J Epidemiol.

[12] Liu S, Kim ED, Wu A, Meyer ML, Cheng S, Hoogeveen RC, Ballantyne CM, Tanaka H, Heiss G, Selvin E, Matsushita K (2019). Central and peripheral pulse wave velocity and subclinical myocardial stress and damage in older adults. PLoS ONE.

[13] Meyer ML, Tanaka H, Palta P, Cheng S, Gouskova N, Aguilar D, Heiss G (2016). Correlates of segmental pulse wave velocity in older adults: the Atherosclerosis Risk in Communities (ARIC) Study. Am J Hypertens.

[14] Manual 2 Home and Field Center Procedures, ARIC Visit 5 and NCS Study Protocol 2013. https://www2.cscc.unc.edu/aric/sites/default/files/public/manuals/Manual%202%20Home%20and%20Field%20Center%20Procedures.pdf.v.

[15] Townsend RR, Wilkinson IB, Schiffrin EL, Avolio AP, Chirinos JA, Cockcroft JR, Heffernan KS, Lakatta EG, McEniery CM, Mitchell GF, Najjar SS, Nichols WW, Urbina EM, Weber T, American Heart Association Council on Hypertension (2015). Recommendations for improving and standardizing vascular research on arterial stiffness: a scientific statement from the American Heart Association. Hypertension.

[16] Tanaka H, Munakata M, Kawano Y, Ohishi M, Shoji T, Sugawara J, Tomiyama H, Yamashina A, Yasuda H, Sawayama T, Ozawa T (2009). Comparison between carotid-femoral and brachial-ankle pulse wave velocity as measures of arterial stiffness. J Hypertens.

[17] Meyer ML, Tanaka H, Palta P, Patel MD, Camplain R, Couper D, Cheng S, Al Qunaibet A, Poon AK, Heiss G (2016). Repeatability of central and peripheral pulse wave velocity measures: the Atherosclerosis Risk in Communities (ARIC) Study. Am J Hypertens.

[18] Parrinello CM, Grams ME, Couper D, Ballantyne CM, Hoogeveen RC, Eckfeldt JH, Selvin E, Coresh J (2015). Recalibration of blood analytes over 25 years in the atherosclerosis risk in communities study: impact of recalibration on chronic kidney disease prevalence and incidence. Clin Chem.

[19] Madan N, Lee AK, Matsushita K, Hoogeveen RC, Ballantyne CM, Selvin E, McEvoy JW (2019). Relation of isolated systolic hypertension and pulse pressure to high-sensitivity cardiac troponin-T and N-terminal pro-B-type natriuretic peptide in older adults (from the Atherosclerosis Risk in Communities Study). Am J Cardiol.

[20] Alonso A, Agarwal SK, Soliman EZ, Ambrose M, Chamberlain AM, Prineas RJ, Folsom AR (2009). Incidence of atrial fibrillation in whites and AfricanAmericans: the Atherosclerosis Risk in Communities (ARIC) study. Am Heart J.

[21] Loehr LR, Rosamond WD, Chang PP, Folsom AR, Chambless LE (2008). Heart failure incidence and survival (from the Atherosclerosis Risk in Communities study). Am J Cardiol.

[22] Rosamond WD, Chambless LE, Folsom AR, Cooper LS, Conwill DE, Clegg L, Wang C-H, Heiss G (1998). Trends in the incidence of myocardial infarction and in mortality due to coronary heart disease, 1987 to 1994. N Engl J Med.

[23] Austin PC, Lee DS, Fine JP (2016). Introduction to the analysis of survival data in the presence of competing risks. Circulation.

[24] Mitchell GF (2014). Arterial stiffness and hypertension: chicken or egg?. Hypertension.

[25] Lim DJ, Ambale-Ventakesh B, Ostovaneh MR, Zghaib T, Ashikaga H, Wu C, Watson KE, Hughes T, Shea S, Heckbert SR, Bluemke DA, Post WS, Lima JAC (2019). Change in left atrial function predicts incident atrial fibrillation: the Multi-Ethnic Study of Atherosclerosis. Eur Heart J Cardiovasc Imaging.

[26] Burstein B, Libby E, Calderone A, Nattel S (2008). Differential behaviors of atrial versus ventricular fibroblasts: a potential role for platelet-derived growth factor in atrial-ventricular remodeling differences. Circulation.

[27] Pandey A, Khan H, Newman AB, Lakatta EG, Forman DE, Butler J, Berry JD (2017). Arterial stiffness and risk of overall heart failure, heart failure with preserved ejection fraction, and heart failure with reduced ejection fraction: the Health ABC Study (health, aging, and body composition). Hypertension.

[28] van Popele NM, Grobbee DE, Bots ML, Asmar R, Topouchian J, Reneman RS, Hoeks AP, van der Kuip DA, Hofman A, Witteman JC (2001). Association between arterial stiffness and atherosclerosis: the Rotterdam Study. Stroke.

[29] Obata Y, Mizogami M, Nyhan D, Berkowitz DE, Steppan J, Barodka V (2017). Pilot study: estimation of stroke volume and cardiac output from pulse wave velocity. PLoS ONE.

[30] Jensen PN, Johnson K, Floyd J, Heckbert SR, Carnahan R, Dublin S (2012). A systematic review of validated methods for identifying atrial fibrillation using administrative data. Pharmacoepidemiol Drug Saf.

