# Supplementary material for: Association of arterial stiffness with incident atrial fibrillation: a cohort study
Source: BMC Cardiovasc Disord. 2021 May 20;21:247. doi: 10.1186/s12872-021-02057-8 (PMC8139144; doi:10.1186/s12872-021-02057-8)
Supplement: Supplementary file 1 — Additional file 1. Table S1. Associations of Carotid-Femoral Pulse Wave Velocity (cfPWV) quartiles with incident AF in the ARIC cohort by sex and race, 20112017. [file 12872_2021_2057_MOESM1_ESM.docx]

**Table S1. Associations of Carotid-Femoral Pulse Wave Velocity (cfPWV) quartiles with incident AF in the ARIC cohort by sex and race, 2011-2017.**

|  | **1^st^ Quartile** | **2^nd^ Quartile** | **3^rd^ Quartile** | **4^th^ Quartile** | **Interaction** |
| --- | --- | --- | --- | --- | --- |
| **cfPWV** | **< 9.5** | **9.5-11.2** | **11.2 -13.2** | **> 13.2** |  |
| **HR (95% confidence intervals)** | | | | | **P- value** |
| Men  N=1535 | 1.27  (0.75,2.14) | ref | 1.49  (0.92, 2.42) | 1.47  (0.89, 2.44) | 0.40 |
| Women  N= 2347 | 1.68  (1.06, 2.65) | ref | 1.74  (1.09, 2.78) | 1.74  (1.07, 2.81) |  |
| Blacks  N=773 | 0.48  (0.13, 1.80) | ref | 1.91  (0.68, 5.3) | 1.56  (0.57, 4.30) | 0.60 |
| Whites  N=3109 | 1.62  (1.13, 2.32) | ref | 1.53  (1.07, 2.18) | 1.50  (1.03, 2.17) |  |

Abbreviations: ARIC: Atherosclerosis Risk in Communities; BMI, body mass index; CI, confidence interval; HR, hazard ratio; NT-proBNP, N-terminal pro B-type natriuretic peptide; ref, reference.

Cox proportional hazards model adjusted for age, sex, center, race, smoking, education, drinking, diabetes mellitus, heart failure myocardial infarction, aspirin, statin, systolic blood pressure, left ventricular ejection fraction, Left atrial volume.
